# Mechanistic roles of microRNAs in hepatocarcinogenesis: A study of thioacetamide with multiple doses and time-points of rats

**DOI:** 10.1038/s41598-017-02798-7

**Published:** 2017-06-08

**Authors:** Harsh Dweep, Yuji Morikawa, Binsheng Gong, Jian Yan, Zhichao Liu, Tao Chen, Halil Bisgin, Wen Zou, Huixiao Hong, Tieliu Shi, Ping Gong, Christina Castro, Takeki Uehara, Yuping Wang, Weida Tong

**Affiliations:** 1 Division of Bioinformatics and Biostatistics, National Center for Toxicological Research, FDA, Jefferson, AR 72079 USA; 2 Informatics & Structure-based Drug Discovery, Discovery Research Laboratories for Innovative Frontier Medicines, Shionogi & Co. Ltd., Toyonaka, Osaka Japan; 3 Division of Genetic and Molecular Toxicology, National Center for Toxicological Research, FDA, Jefferson, AR 72079 USA; 4 University of North Carolina at Charlotte, 9201 University City Blvd, Charlotte, NC 28223 USA; 5 Department of Computer Science, Engineering, and Physics, University of Michigan-Flint, 303 East Kearsley Street, Flint, MI 48502 USA; 6 Center for Bioinformatics and Computational Biology, and the Institute of Biomedical Sciences, College of Life Science, East China Normal University, Shanghai, China; 7 Environmental Laboratory, U.S. Army Engineer Research and Development Center, 3909 Halls Ferry Road, Vicksburg, MS 39180 USA; 8 Global Project Management Department, Shionogi & Co., Ltd., Osaka, Japan

## Abstract

Environmental chemicals exposure is one of the primary factors for liver toxicity and hepatocarcinoma. Thioacetamide (TAA) is a well-known hepatotoxicant and could be a liver carcinogen in humans. The discovery of early and sensitive microRNA (miRNA) biomarkers in liver injury and tumor progression could improve cancer diagnosis, prognosis, and management. To study this, we performed next generation sequencing of the livers of Sprague-Dawley rats treated with TAA at three doses (4.5, 15 and 45 mg/kg) and four time points (3-, 7-, 14- and 28-days). Overall, 330 unique differentially expressed miRNAs (DEMs) were identified in the entire TAA-treatment course. Of these, 129 DEMs were found significantly enriched for the “liver cancer” annotation. These results were further complemented by pathway analysis (Molecular Mechanisms of Cancer, p53-, TGF-β-, MAPK- and Wnt-signaling). Two miRNAs (rno-miR-34a-5p and rno-miR-455-3p) out of 48 overlapping DEMs were identified to be early and sensitive biomarkers for TAA-induced hepatocarcinogenicity. We have shown significant regulatory associations between DEMs and TAA-induced liver carcinogenesis at an earlier stage than histopathological features. Most importantly, miR-34a-5p is the most suitable early and sensitive biomarker for TAA-induced hepatocarcinogenesis due to its consistent elevation during the entire treatment course.

## Introduction

Many environmental chemicals and pharmaceuticals can cause liver toxicity and cancer^1, 2^. The majority of these so-called chemical carcinogens act through covalent binding to the DNA (DNA adduct formation), thus known as genotoxic carcinogens^3–5^. Genotoxic carcinogens are commonly determined using the genotoxic battery of assays (e.g., Ames test). Of note, many studies are being developed to improve the accuracy and specificity of these assays^6, 7^. However, some chemical carcinogens act through non-genotoxic mechanisms, known as non-genotoxic carcinogens (NGTCs)^8, 9^. NGTCs are commonly determined by rodent two-year bioassays^10–12^, which are time-consuming, expensive and require a lot of animals. Most unfortunately, the two-year bioassays sometimes fail to identify NGTCs^13^. Many argue that extensive research should be placed on developing new biomarkers and assay models to replace two-year bioassays through enhanced understanding of mechanism underlying NGTCs^14^. Thioacetamide (TAA) is a well-known NGTCs, which is a possible human carcinogen^15^. When administrated orally, it is reported to induce hepatocellular adenomas in rats^16^. In this study, we designed a toxicogenomics experiment involving multiple doses and treatment durations to study microRNA (miRNA) expression of TAA-induced carcinogenicity, thus further our knowledge to NGTCs.

Toxicogenomics has been extensively used for the study of environmental carcinogens^17^. It has been well established that gene expression profiles are associated with carcinogenicity and thus genomics approaches have been used for cancer risk assessment^18, 19^. Many studies have attempted to classify unknown carcinogens based on gene expression profiles^19–21^. MiRNAs, a family of small noncoding RNAs, have been recognized as key regulators for cancer development and progression through regulatory function over multiple cancer-related target genes^22, 23^. They play an important role in regulating mRNAs expression as well as protein translation in both states of physiology and cancer development. In mammals, miRNAs are predicted to control the activity of approximately 30% of all protein-coding genes and have been shown to participate in the regulation of almost every cellular process^24, 25^. Importantly, miRNAs have been observed to be tissue-specific^26^. Distinct miRNA profiles can be assigned to various types of tumors, serving as molecular signatures in cancer diagnostics and prognostics. Recent technological advances have offered several options for miRNA detection and assessment^27^. Among them, next generation sequencing (NGS) has gained an advantage for miRNA profiling, the discovery of novel miRNAs, and isoforms of established miRNAs^28^.

**Figure 1:**
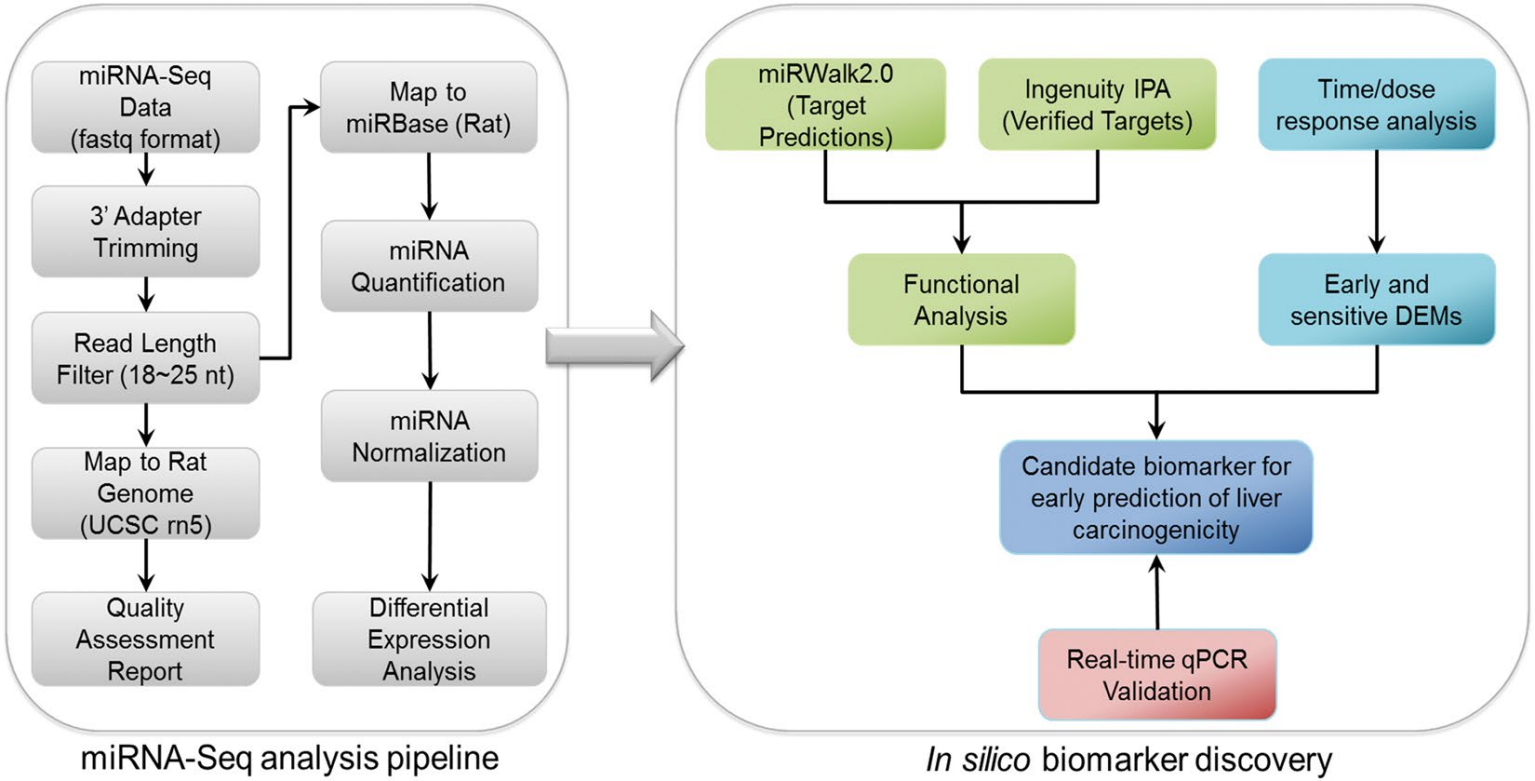
A schematic illustration of the workflow used in the present study to elucidate differentially expressed miRNAs during TAA-induced hepatocarcinogenesis. This workflow can be broadly divided into two sections: miRNA-Seq analysis pipeline and *in silico* biomarker discovery. In miRNA-Seq analysis pipeline, briefly, the adaptor sequence was trimmed from the fastaq files obtained from Illumina HiSeq2000 platform and then reads with a length between 18 to 25 bases were selected to map to the Rattus norvegicus (rn5) genome. Quality control and assessment were carried out on the selected dataset. In a next step, reads were mapped against miRBase (rat) and quantification and normalization were performed to obtain DEMs. In the second section, functional association (using miRWalk2.0 and Ingenuity IPA) and time/dose response analyses were conducted to identify early and sensitive DEMs and their possible regulatory signaling in TAA-induced liver carcinogenicity. Thereafter, qPCR experiments were performed to validate potential biomarker. Information about the programs and their parameters used in this workflow is given in detailed under the Methods section.

In this study, we examined the change in the expression profile of miRNAs over multiple doses and time-points of treatment with TAA using NGS technology (miRNA sequencing or miRNA-seq). A new integrative bioinformatics workflow was applied to identify differentially expressed miRNAs (DEMs) and their possible regulatory roles within the signaling cascades which could be involved in TAA-induced hepatocarcinogenicity (Fig. 1). To the best of our knowledge, this is so far the very first toxicological study conducted to investigate DEMs at various doses and time points for environmental chemical exposure. We found that the identified DEMs were significantly overrepresented for the liver cancer and many pathways were related to TAA-associated carcinogenicity. Importantly, the expression levels of two DEMs (i.e., rno-miR-34a-5p and rno-miR-455-3p) were found to be changed with almost all treatment conditions. Especially, rno-miR-34a-5p was the most upregulated DEMs throughout the entire treatment, suggesting its potential to be utilized as early and sensitive biomarkers to detect TAA-modulated carcinogenicity. Surprisingly, miR-122, which is a commonly studied circulating miRNA biomarker for liver injury, was not differentially expressed according to neither dose nor time point in the liver tissues treated with TAA.

## Results

The workflow depicted in Fig. 1 identified 330 DEMs using the cutoff of fold change >1.5 and p-value < 0.05 in TAA-treated rats compared to the control group across all the treatment conditions (Table S1). The results obtained from this workflow are summarized in Table 1. Further investigations were performed to detect early and sensitive biomarkers based on these 330 DEMs and to decipher their roles in liver cancer and related biological signaling pathways. The results of these analyses are explained in the sections below.

**Table 1:** Summary of the results obtained from the bioinformatics workflow.

Treatment	Upregulated (N)	Downregulated (N)
*Distribution of 330 DEMs*		
Low dose	152	174
Middle dose	165	153
High dose	134	137
*Total DEMs* (*unique*)	176	211
*Common DEMs*	48	
*Dose- and time-dependent DEMs*	7	
*Dose-dependent DEMs*	39	
*Time-dependent DEMs*	2	
*Diseases or functional association*	22	

### Liver cancer predicted by miRNA profiling

**Figure 2:**
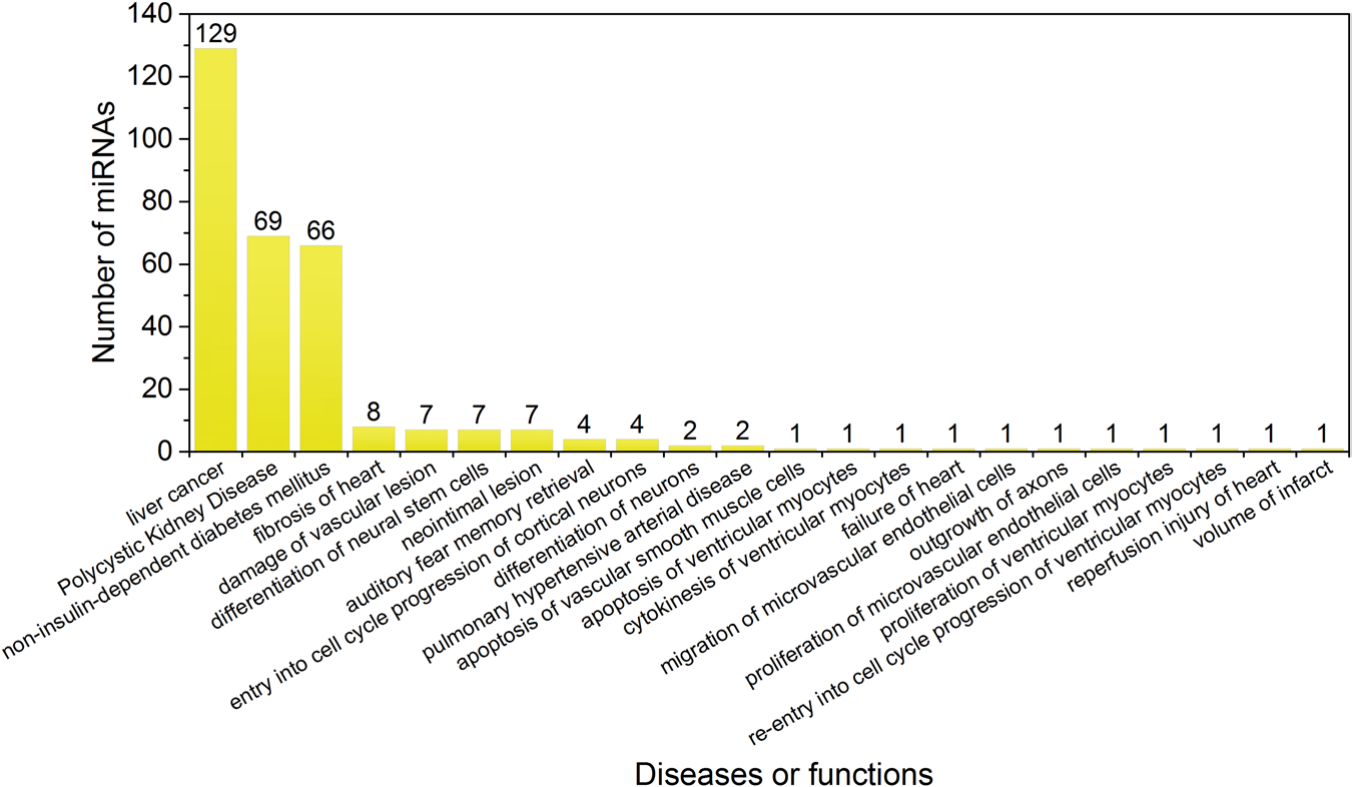
Disease enrichment analysis of DEMs. The liver cancer is predicted as highly significantly enriched with the maximum number of DEMs during disease/function overrepresentation analysis using IPA software. The x-axis depicts diseases/functions, whereas, the y-axis represents the number of DEMs mapped to diseases or functions.

A disease/function enrichment analysis was conducted using Ingenuity Pathway Analysis (IPA) to identify the pathophysiological association of DEMs during three different doses and four time intervals (12 treatment conditions), which resulted in 22 diseases or functions significantly enriched (Table S2). The top three were liver cancer, polycystic kidney disease, and non-insulin-dependent diabetes mellitus. The “liver cancer” was the most significantly enriched term (with the largest number of DEMs i.e. n = 129) amongst the top three diseases for all 12 treatment conditions (Fig. 2). Moreover, the liver cancer was found significantly enriched in the low dose conditions of all the treatment durations. For example, in total, 54 DEMs were mapped to liver cancer annotation at the low doses for 3-d (p = 1.36e-23), 7-d (p = 3.67e-18), 14-d (p = 6.38e-23) and 28-d (p = 1.15e-13). On the other hand, the liver tissues of rats treated with low dose of TAA were found to have minimal nuclear alternation in their livers (http://virtualslide.nibiohn.go.jp/Authenticate3.php?image_id=46720; http://virtualslide.nibiohn.go.jp/Authenticate3.php?image_id=46727) only at 28 day, but no single rat was found to have noticeable histopathological features in the liver at 3-, 7 and 14-d (Figure S1). These results suggest that miRNA profiling has the potential to detect early biomarkers (miRNAs signature) during TAA-induced liver carcinogenicity at low exposure levels than histopathological features.

**Figure 3:**
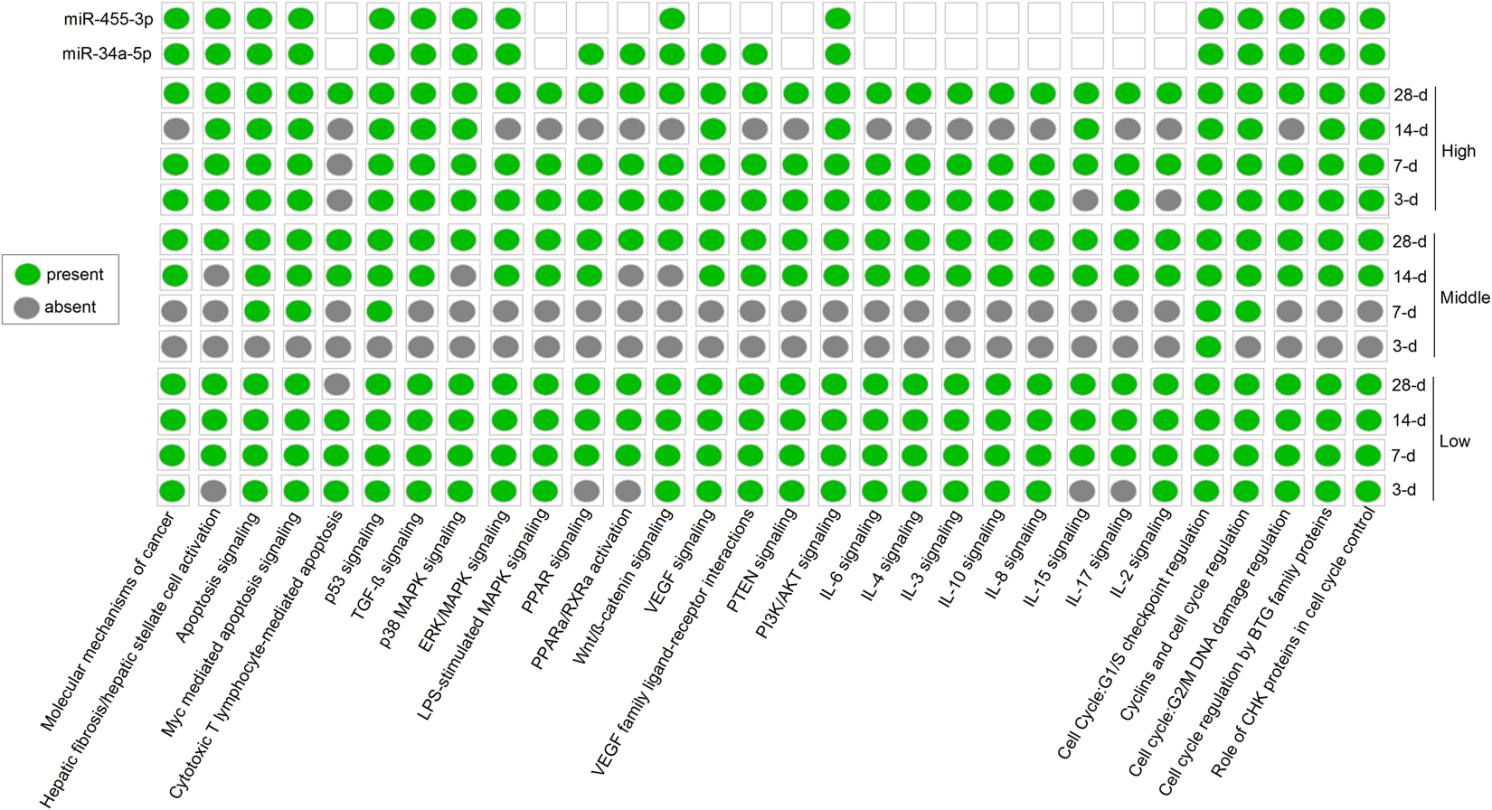
Heat map of most relevant pathways on DEMs. This heat map depicts the most significant and highly relevant pathways obtained using experimentally verified targets of differentially regulated miRNAs (DEMs). Information on different doses and time intervals is shown on the right side and relevant signaling are shown at the bottom. Information on two potential early and sensitive miRNA biomarkers (miR-34a-5p and miR-455-3p) and their associated signaling is given on the top two rows. The top two rows depict whether a given miRNA is predicted to regulate a selected pathway or not. The “green” and “gray” colors indicate either a pathway is present or absent respectively.

To further support our findings, we retrieved the experimentally observed target genes for the DEMs identified in each treatment condition from IPA database and performed pathway enrichment analysis. We then ranked the enriched pathways by the statistical significance for all the conditions. As shown in Fig. 3 (and Table S3 with a full list of enriched pathways), “Molecular Mechanisms of Cancer” is the most related and highly significantly enriched pathway in 9 of the 12 conditions. Other cancer-, immune- and hepatotoxicity-related pathways were also identified to be related to most TAA treatment conditions, such as “p53 signaling”, “TGF-β signaling”, “MAPK signaling”, “PPARα/RXRα activation”, “Hepatic fibrosis/Hepatic stellate cell activation” and “Wnt signaling”. Notably, *miR-455-3p* and *miR-34a-5p* were involved most of the cancer-related functional pathways. Furthermore, the pathway enrichment analysis of putative target genes of the DEMs yielded similar signaling cascades (highly significantly enriched) including cancer, immune, cell cycle, apoptosis, p53 signaling, Wnt signaling, MAPK signaling and other relevant pathways which further complement the functional findings of the DEMs using IPA-based methods, suggesting a potential role of miRNA-mediated regulation during TAA treatment (Tables S4 and S5). Taken together, we were able to relate particular treatment group that had up- and down-regulated miRNAs to each significant function (such as mechanisms of cancer, p53 signaling, and metabolic pathways) and annotation in diseases (e.g., liver cancer). A complete list of these DEMs and their toxicological functions are presented in Tables S4 and S5.

### Early and sensitive biomarkers detection by measuring changes in miRNA expression profiling

**Figure 4:**
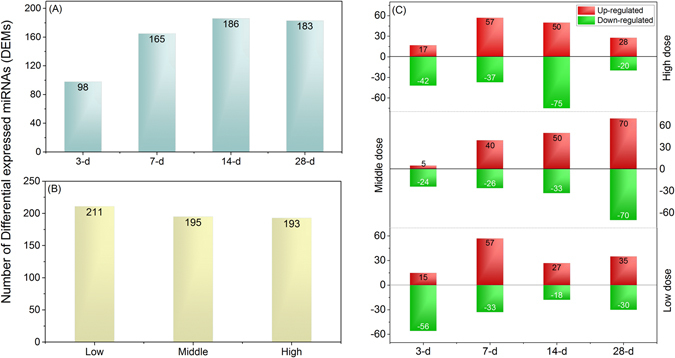
Distribution of DEMs during across all treatment conditions. (**A**) describes an increase in the number of DEMs across four time intervals, (**B**) shows a decreasing in the count of DEMs with increasing dosing from low to high and (**C**) represents the distribution of up-and down-regulated miRNAs at three different doses with four time intervals. X and Y axes represent days and number of DEMs observed during TAA treatment. Red and green color bar plots denote up- and down-regulated miRNAs respectively. From bottom to top, the maximum number of downregulated miRNAs (n = 174; green bars) are observed with low dose compared to middle (n = 153) and high (n = 137) doses, whereas, the highest number of upregulated miRNAs are observed with middle dose (n = 165). Of note, an increase in the number of up- and down-regulated miRNAs is determined at middle dose with all four time points (middle bar plot).

The above findings demonstrated that hundreds of miRNAs were differentially expressed across the entire course of TAA treatments even at low dose, suggesting that these tiny regulators could be involved in TAA-induced liver carcinogenicity. Thus miRNAs signature could serve as early and sensitive biomarkers for the diagnostic and prognostic of liver carcinogenicity. For that, DEMs were grouped into time points (3-d, 7-d, 14-d and 28-d) and doses (low, middle and high). The number of DEMs was increased in the case of time points group i.e. 3-d (n = 98), 7-d (n = 165), 14-d (n = 186) and 28-d (n = 183) (Fig. 4A). However, with increasing dosing (low to high), a decrease in the number of DEMs was observed i.e. low dose (n = 211), middle dose (n = 195) and high dose (n = 193) (Fig. 4B). Further, DEMs were compiled into up- and down-regulated classes (Fig. 4C). In total, 176 and 211 unique miRNAs were up- and down-regulated at more than one-time point studied, indicating more miRNAs downregulated than upregulated. At 3-d with three doses, the number of downregulated miRNAs were higher than upregulated ones, whereas, more miRNAs were found upregulated compared to downregulated at 7-d with different doses (Fig. 4C). Interestingly, a continuous increase in the count of up- and down-regulated miRNAs was observed at middle dose treatments (Fig. 4C). On further analysis, five out of 12 treatment conditions were found to have a higher number of DEMs (larger than the mean value of n = 77). Of these five treatments, the maximum number of DEMs (n = 140) was determined with the middle dose at 28-d, whereas, 125, 94, and 90 and 83 DEMs were estimated with low (14-d and 7-d) and high doses (at 7-d) (Fig. 4C). On the other hand, the number of DEMs for low dose at 3-d was much higher (n = 59) when compared to low (28-d), middle (3-d) and high doses (14-d). These observations indicate that many miRNAs expressions occur early in the treatment and a few of them consistently express across the entire course of treatments, suggesting that these DEMs could be candidates for an early biomarker for NGTCs.

**Figure 5:**
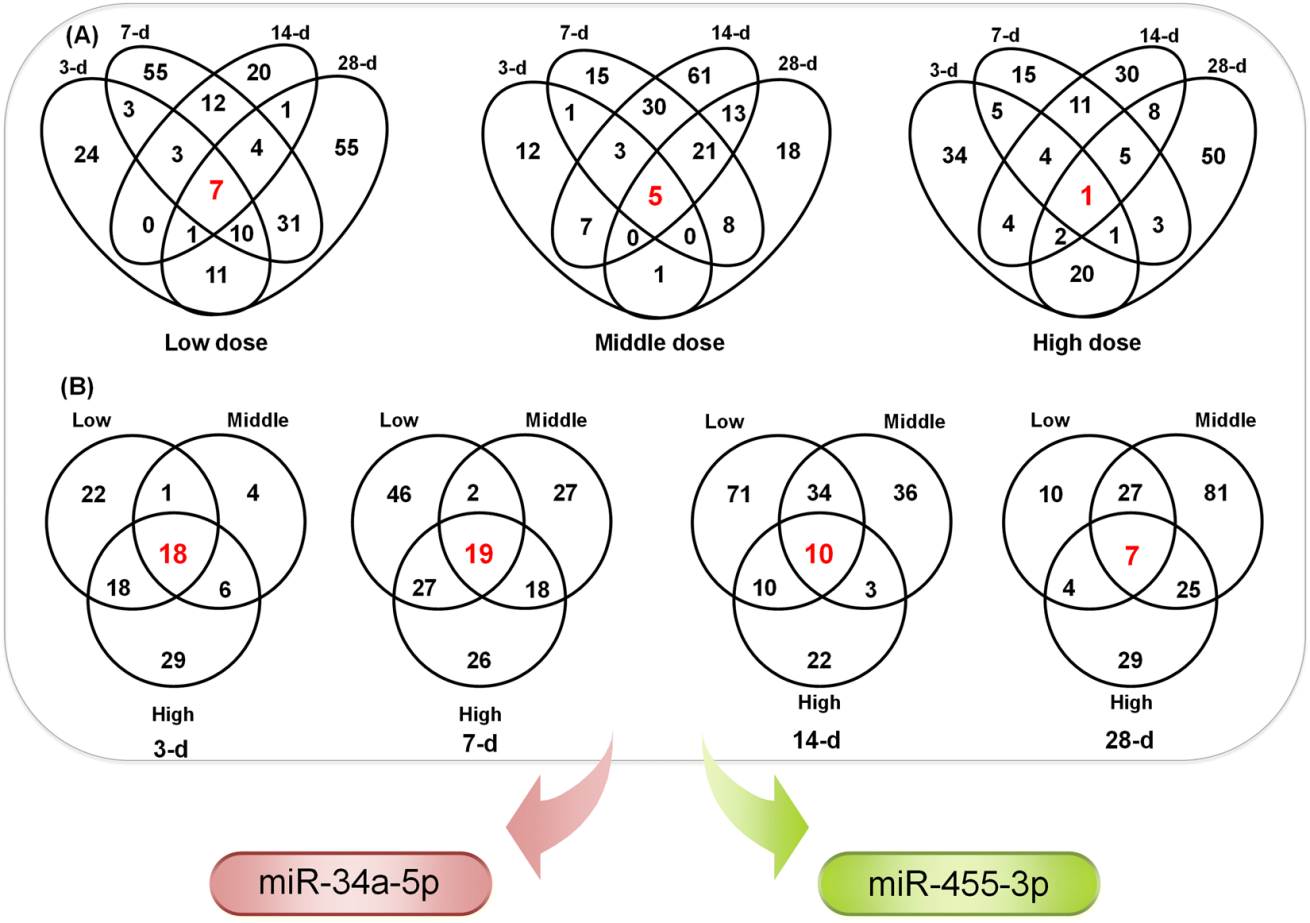
Venn diagrams of DEMs during different time points and doses. (**A**) depicts DEMs during low, middle and high doses with different time points, respectively and (**B**) describes DEMs during 3-, 7-, 14- and 28-d with three different doses (low, middle and high). Two potential biomarkers (miR-34a-5p and miR-455-3p) out of 48 overlapping DEMs are found common across all the treatments.

**Figure 6:**
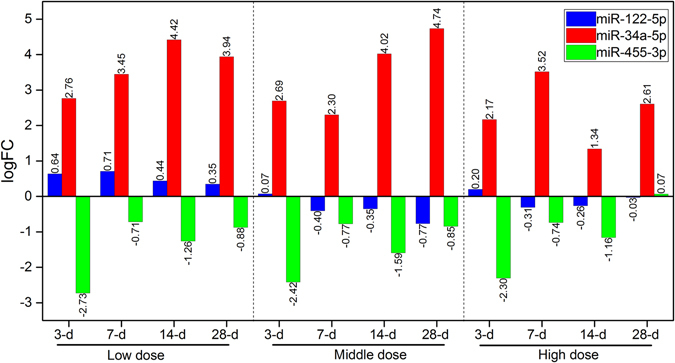
Overview of expression patterns of 2 early and sensitive miRNAs. From the top, the logFC expression of rno-miR-122-5p is not significantly differentially expressed when compared with control animals. Rno-miR-34a-5p is highly significantly and consistently overexpressed among different doses and time points, suggesting the potential of this miRNA to be considered as an early and sensitive biomarker for monitoring TAA-induced hepatocarcinogenesis during low to high exposures. At the bottom of this figure, the higher expression values of rno-miR-455-3p in the control compared to treated samples, as a result, this miRNA is identified as significantly downregulated with all three doses and all four time points. The highest down-regulation of this miRNA is observed at 3-d with all three doses. The absolute fold change values are used to plot this figure.

Figure 5 is Venn diagrams for overlapped DEMs among different treatment conditions along dose and time. In general, the common DEMs among time points (Fig. 5A) were much less compared to those among doses (Fig. 5B). Venn diagrams for time-dependent miRNAs across different doses yielded 7, 5 and one common DEMs for low, middle and high doses respectively (Fig. 5A). In the case of dose-dependent miRNAs (Fig. 5B) at four time- points, 18, 19, 10 and 7 DEMs were common during low, middle and high doses, indicating a decrease in the number of DEMs with an increasing pattern of dosing (low-middle-high). On the other hand, an increasing in the number of DEMs (those confined to middle dose) was observed for all four time points (Fig. 5B). A similar observation was also noticed for DEMs that are only found with low and high doses at 3-d, 7-d, and 14-d. After dose- and time-dependent comparison analysis, a total of 48 DEMs was selected to investigate further whether the fold-change values of these overlapping DEMs follow any increasing, decreasing or stable expression trend. Two DEMs were found to be both dose and time responsive. Amongst them, rno-miR-34a-5p, an upregulated DEM, is the most responsive candidate with more than 4 fold change in most of the treatment conditions studied (Fig. 6). It is interesting to note that a decreasing trend of the fold change for this DEM was observed with all three doses at 7-d and 14-d. Moreover, an increasing in the expression fold change values of rno-miR-34a-5p was observed with low (except 28-d) and middle doses (all four time points), however, for high dose, a mixed expression trend was observed. These observations may suggest that the rno-miR-34a-5p tends to give up its regulatory function at a higher dose. Additionally, the second arm of miR-34a (i.e. rno-miR-34a-3p) was also upregulated with more than 3-fold in 7 out of 12 treatment conditions. On the other hand, rno-miR-455-3p, a downregulated DEM, was observed in 11 out of 12 treatment conditions (excluding high dose at 28-d).

Taken together, our findings indicated that miR-34a-5p and miR-455-3p could be potentially considered as early and sensitive biomarkers for TAA-induced hepatocarcinogenicity. Thus, further analyses were carried out on these two miRNAs.

### Expression of miR-34a-5p, miR-455-3p and miR-122

The miR-34a-5p and miR-455-3p were selected to investigate their expression changes during TAA treatments. These two miRNAs were chosen due to their dose- and time-dependent trends as well as their fold changes (Fig. 6). Another miRNA, miR-122, was also selected to measure its expression because many studies have considered miR-122 as an important non-invasive biomarker for liver injury in blood. Therefore, to investigate the dynamic changes of these three miRNAs, the upper quantile normalized and logarithm of fold change (logFC) values were plotted for all the samples (Fig. 6). The rno-miR-122 was found as one of the top ten most abundant miRNAs in the livers across all the treatment. However, the expression of this miRNA was nonsignificant in dose as well as time groups during differential expression analysis (Fig. 6), suggesting that miR-122 may not be directly involved in the mechanism of TAA-mediated hepatotoxicity in liver tissue. On the contrary, expression of rno-miR-34a-5p was significantly increased (logFC > 1.5 to 4.7) after treatment and consistently presented over time and doses (Fig. 6). However, the expression of miR-455-3p was significantly downregulated across the entire course of TAA treatments (except 28-d with high dose). These observations showed their time- and dose-dependent trends, indicating their key involvement in the regulation of liver toxicity after TAA treatment.

Significantly increased expression of *rno-miR-34a-5p* was identified across all doses following 7-d, 14-d, and 28-d of treatments. The highest elevation in the expression was measured at 28-d with middle dose (Fig. 6). *Rno-miR-34a-5p* was detected to be very low in the baseline read count over the full-time course in the control group. The abundance of this miRNA significantly increased in all the treatment groups compared to the control animals across all the time points. On the other hand, rno-miR-455-3p was comparatively higher in the controls than treated samples. After statistical analysis of differential expression, the highest down-regulation of this miRNA was observed at 3-d with all three doses and at 7-d, 14-d, and 28-d, a stable downregulation expression pattern was observed with low, middle and high doses. Functional annotation of this two dose- and time-dependent DEMs demonstrated their active involvement in the hepatocellular carcinoma (HCC) network, as well as other liver disease networks. Previous investigations have already been demonstrated a key role of miR-34a in liver cancer, however, so far there is no single study exists which have studied its expression and role in TAA-induced hepatocarcinogenicity along different doses and time intervals. In addition to miR-34a-5p, we found miR-455-3p which can potentially be used as an early and sensitive biomarker for the detection of liver injury and pre HCC.

To further evaluate the correlation between these two DEMs and cancer progression, we carried out a regression analysis between the histopathological features and the expression patterns of miR-34a-5p, and miR-455-3p during TAA exposures. In case of miR-34a-5p, the cellular infiltration, eosinophilic change, fibrosis, bile duct proliferation and single cell necrosis features were found to cover more than 0.5 AUC (area under curve). Most importantly, the single cell necrosis was found to have the maximum value i.e. 0.75. On the other hand, the nuclear alteration and cellular loci classifiers out of 6 histopathological features were found to obtain the maximum AUC values for miR-455-3p (Figure S1). These features are indicative of loss or distortion of normal liver architecture, but mostly with high dose exposures. To validate the findings from miRNA-seq, we therefore measured the expression levels of miR-34a-5p at high dose exposures of TAA using real-time qPCR (RT-qPCR). *Rno-miR-34a-5p* was selected due to its consistent upregulation across the entire course of treatments as well as its critical function as tumor suppressor in HCC. PCR results demonstrated that expression of *rno-miR-34a-5p* was significantly increased across three time points (p < 0.05 at 7-d, and 14-d, p < 0.005 at 28-d) treatments compared to the control group (Figure S2). Furthermore, the trend of miRNAs deregulation detected from qPCR and NGS are consistent, indicating the findings from miRNA-seq were reliable.

## Discussion

TAA is a potent NGTC and has been confirmed to be a carcinogen in animal studies^29, 30^ and possible human carcinogen, but the lack of information about its carcinogenicity and effective approach to characterize its carcinogenesis. Being key regulators of multiple biological functions as well as potential promising biomarkers for diseases, miRNAs have been extensively studied for the past decades^31–33^. In this study, we profiled miRNAs from rat livers treated with a TAA at multiple doses over four time points to characterize the dynamic changes of miRNAs expression and explore the mechanistic roles of miRNAs resulting in the carcinogenesis. Through comprehensive data quality assessment, bioinformatics pipeline development and analysis of miRNAs sequencing data analysis, differentially expressed miRNAs at various doses or time points were identified. Additionally, scatterplot analyses were performed to estimate the consistency (correlation coefficient) of the expression data sets among three replicates (rats) at different doses and time points (Figs 3, 4 and 5).

Time-course and dose-dependent data are the two most important parameters for toxicological responses which are either transient, continuous or are delay responses. Upon comparing, DEMs observed with low-dose at 3-d were more than middle dose, however, there is neither dose- nor time-dependent patterns were observed on the number of deregulated miRNAs at a low dose and 3-d treatments. Most of the DEMs only appeared transit deregulation at a low dose and short duration of treatments may function as adaptive responses to the procedures or stress of treatment. Similar time-dependent observations have been reported previously^34^. In the case of middle dose treatment compared with the control group, the number of both up- and down-regulated miRNAs appeared in a time-dependent manner indicating the gradually massive involvements of miRNAs along the duration of mild exposure which might be taken into consideration for dose selection of carcinogenic study. For a high dose, the highest numbers of up- and down-regulated miRNAs were observed at 14-d and most of those DEMs identified were consistently differentially expressed at 28-d high dose group. These findings may be an indicator for the best window for a sampling of the maximum tolerated dose (MTD) exposure while conducting miRNAs experiments for carcinogenesis assessment. Following functional analysis showed that those consistently deregulated miRNAs are relevance towards toxicity/carcinogenicity assessment, indicating that deregulation of miRNAs expression appeared earlier that the histopathological features. In addition, there is no consistent proportion of up- and the down-regulated miRNAs number was observed across the entire course of treatments. Previously, it has been suggested that the downregulation of miRNAs may occur by putative oncogenes^35^. Conversely, upregulated miRNAs may be tumor suppressors^36, 37^. Therefore, oncogenic and suppressive miRNAs were roughly close-fought with the current treatment conditions.

Dysregulation of miRNAs has already been linked to various pathological processes including cancer. In the present study, we identified 129 DEMs (including up- and down-regulated candidates obtained with all three doses) that are primarily highly significantly enriched in liver cancer using a disease enrichment overrepresentation analysis. To further support these observations, the pathway enrichment analysis of deregulated miRNAs identified at each dose and time point of TAA treatment found “Molecular Mechanisms of Cancer” as the most related and significant pathway in 9 of the 12 TAA treatment conditions. Moreover, immune and hepatotoxicity related pathways were also identified to be related to most TAA treatment conditions, such as “p53 signaling”, “TGF-β signaling”, “MAPK signaling”, “PPARα/RXRα activation”, “Hepatic fibrosis/Hepatic stellate cell activation” and “Wnt signaling”. Similar findings were confirmed with the different approach for pathway enrichment analysis using putative target genes of DEMs using miRWalk2.0. The p53 signaling pathway is considered as an indicator of DNA damage in response to stress. Activation of this pathway was proposed to disrupt the fidelity of DNA replication and cell division^38, 39^. Significantly enriched p53 pathways after treatment of TAA at early stage indicates the activation of DNA repair and promote cellular proliferation.

Using Venn diagrams analysis, two potential DEMs were identified as commonly expressed during almost all the treatment conditions. Notably, a significant increase in the expression of *rno-miR-34a-5p* was identified across all doses following 7-d, 14-d and 28-d of treatment. While the expression of rno-miR-455-3p was decreased with an increase in the dose as well as followed a downregulation with almost all the time points (excluding high dose at 28-d). In a previous study, miR-455-3p has been observed as significantly downregulated in poorly differentiated HCC. Also, functional analysis (including association disease investigation) demonstrated significant enrichment in the cancer network. Previously, miR-34a has been reported to play important roles in the pathogenesis of several human diseases, including liver carcinogenesis^40, 41^. As a well-known tumor suppressor, the expression of miR-34a was significantly inhibited in patients with HCC in clinical studies^41, 42^. Thus, chemicals showing the capability of the restoration of miR-34a function could be proposed as a potential therapeutic agent for HCC. We found that *rno-miR-34a-5p* was consistently induced after treatment of TAA at a low dose and at 3-d time point demonstrating that anti-cancer activity of the biological system was triggered at the early stage of dosing which indicated that miRNAs of *rno-miR-34a-5p* might be a potential biomarker to characterize the carcinogenesis for environmental carcinogens in a short term experiment. We further compared miRNA expression profiling with the histopathological features that are carefully determined by expert pathologists on the rat liver tissues treated with 3 doses (low, middle and high) and 4 time points (3-d, 7-d, 14-d, and 28-d). As a result, the minimal nuclear alteration was observed in the rat liver sections treated with low dose at 28-d, but no single histopathological features were detected at 3-, 7- and 14-d with low dose of TAA. On the other hand, the early signal signatures of liver cancer was successfully predicted by miRNA profiling with low dose exposures at 3-d (p = 1.36e-23), 7-d (p = 3.67e-18), 14-d (p = 6.38e-23) and 28-d (p = 1.15e-13). These findings suggest that toxicogenomics has a great potential to detect early signals of carcinogenic exposures and is a substantial tool for toxicological research. Furthermore, our regression analysis between the expression data and histopathological features (Figure S1) depicts a strong correlation among two DEMs and cancer progression. For example, the single cell necrosis was found to obtain the maximum (AUC = 0.75) for miR-34a-5p. Necrosis is a hallmark of carcinogens-induced liver injury and thought to be an intensive form of inflammatory mode of cell death as compared to apoptosis. Moreover, it has been thought of as an unregulated form of cell death, with multiple simultaneous cellular events culminating in cell swelling and plasma membrane rupture. These necrotic processes include loss of ion homeostasis causing cell swelling, increases in cellular free calcium, activation of diverse proteases and phospholipases, and loss of mitochondrial integrity^43^. Similar, signaling cascades such as Wnt, proteolysis, metabolism, MAPK, cell cycle and apoptosis were predicted to be significantly regulated by miR-34a-5p. In case of miR-455-3p, the nuclear alteration and cellular loci classifiers cover the maximum AUC values (Figure S1). These features are indicative of loss or distortion of normal liver architecture. Our pathway enrichment analysis of miR-455-3p targets also found several relevant biological signaling pathways including pathways in cancer, focal adhesion, pancreatic cancer, glioma, basal cell carcinoma, and Mapk-, Wnt- and mTOR-signaling, suggesting alteration of liver architecture.

On the other hand, miR-122 has been extensively studied and keen interest was expressed as a promising circulating biomarker in liver disease^44–46^. Nonetheless, experimental affirmation for its mechanistic roles in liver disease and hepatocarcinogenesis is lacking. Even though abundant *rno-miR-122* was consistently expressed over each treatment condition in this study, there was no differential expression of *rno-miR-122* compared to the concurrent control animals at neither time nor dose, indicating a lack of the relevance to effects associated with the treatment. Taking the consideration of lack of mechanistic relevance to the treatment regardless the abundant expression in the rat livers of *rno-miR-122*, it could serve as a house-keeping miRNA for the liver.

Notably, *rno-miR-34a-5p* and *rno-miR-455-3p* were detected differentially expressed following all time-points and doses compared to the control group (Fig. 6), indicating the significant relevance of the expression of both miRNAs to the treatments. These miRNAs were also predicted to regulate cancer, immune and other relevant signaling cascades (Fig. 3). Furthermore, the results obtained from qPCR experiments confirmed a significant elevation in the expression of miR-34a-5p after treatments and these outcomes showed a similar pattern of miRNAs expression with results from NGS technology (Figure S2). However, further experiments including the qPCR validation of these DEMs during different dose exposures are needed to establish these findings. In the future, we will expand this project by introducing gene expression profiling to defining a landscape of genes, transcription factors (TFs), miRNAs and their signaling pathways, and to identifying the key interactions among genes-DEMs-TFs to reveal the mechanistic relevance of these functional loops and will validate potential candidates.

## Conclusions

Our meta-analysis approach (bioinformatics-, and statistical analysis and qPCR validation) suggest that miRNAs play critical role in the mechanisms underlying hepatocarcinogenesis after TAA exposure. Significant deregulation of miRNAs detected at short-term exposure with dose/time-dependent manner, demonstrating that miRNAs profiling may be an indicator to characterize the carcinogenicity for environmental carcinogens and apply for risk assessment. Furthermore, we have shown significant regulatory associations between two DEMs expression and TAA-induced liver carcinogenesis at an earlier stage than histopathological features. These two miRNAs could be considered as potential biomarkers for the detection of TAA-induced liver carcinogenesis. Combing through the unique natures of miRNAs, such as tissue-specific, stable in body fluids and conserved cross-species; miRNAs profiling analysis could be a potential approach for cancer risk assessment in humans.

## Methods

### Animal treatment

The animal study was conducted as described previously^47^. Briefly, male Sprague-Dawley rats were consecutively administered with TAA once daily at doses of 4.5 (low dose), 15 (middle dose), or 45 (high dose) mg/kg for 3, 7, 14, and 28 days (referred as 3-d, 7-d, 14-d and 28-d hereafter) with the time-matched control group receiving vehicle alone. All animal studies were conducted by the Toxicogenomics Project (TGP) group after obtaining approval from the Ethics Review Committee for Animal Experimentation of the National Institute of Health Sciences, Japan. All experimental protocols were approved by the National Center for Toxicological Research (NCTR), U.S. Food and Drug Administration (FDA). All the methods were carried out in accordance with the approved guidelines. The animals were sacrificed 24 hours after the last dosing day and liver samples were isolated for miRNA-seq analysis as described below. At each treatment condition (the combination of dose and time point), five rats were applied, of which three were used for miRNA profiling. There were twelve treatment conditions in the experiments (=3 doses × 4-time points).

### RNA extraction from rat livers

Total RNA was isolated from approximately 15 mg liver sections using miRVana^*TM*^ isolation kit (Ambion® ThermoFisher Scientific, Grand Island, NY, USA) according to manufacturer’s protocol. The yield of the extracted RNA was determined spectrophotometrically by measuring the optical density at 260 nm (Nanodrop-1000, Thermo Scientific, Wilmington, DE, USA). The quality of RNA samples was characterized with Agilent BioAnalyzer (Agilent Technologies, Santa, Clara, CA, USA) using the RNA6000 Nano Chip (Agilent Technologies). All the RNA samples had RNA integrity numbers (RINs) greater than 8.0 with an average RIN of 9.2.

### Library construction and miRNA-seq

miRNAs were profiled using Illumina HiSeq-2000 platform for three biological replicates of each treatment condition. It was performed by the service provider (Microarray Core Facility, the University of Texas at Southwestern, Dallas, TX, USA). Briefly, 1 µg of extracted total RNA was used for miRNA-seq library preparation using Illumina TruSeq small RNA sample preparation kit. Each RNA sample was processed individually by adding special bar-coded adaptors and reverse transcriptase and amplified. The quantity and quality of each library were measured by Picogreen DNA quantification and Bioanalyzer high sensitive Pico chips (Agilent Technologies). Forty-eight barcoded libraries were then pooled by mixing equimolar of DNA. The pooled libraries were then loaded onto 6% high-resolution PAGE gel for separation of miRNA from other RNAs. The bands with the molecular weight between 145–160 bp were excised into recovery tubes for gel purification. The recovered barcoded libraries were then sequenced on the Illumina HiSeq2000 platform with single end base pair reads. The sequencing data were de-multiplexed and transformed to fastq data files. The read depths for each sample were about 10 million. All of the raw miRNA sequencing data discussed in this study have been deposited in NCBI’s Gene Expression Omnibus (GEO) with accession number GSE87446.

### Processing of miRNAs sequencing data

As miRNAs are short (approximately 18–25 nucleotides long) and the routine sequencing generally has a read length of 50 bps, the adaptor sequence was trimmed. Read with length less than 18 bases or more than 25 bases after trimming (as default) were discarded. The further quality check was performed after trimming to evaluate the distribution of read length, which was anticipated to be centered at 21 bases for a good miRNA-seq library preparation. Schematic workflow for this study is depicted in Fig. 1. The quality of samples was assessed by FastQC tool and all the samples passed the Phred quality score cutoff of 30 (99.9% base call accuracy). All miRNA reads were then mapped to the Rattus norvegicus (rn5) genome to verify indeed all miRNAs are from rats.

Quality control and assessment for the dataset are showed in Figure S6. In summary, read depth span from 5 to 15 averaging about 10 million for each sequencing run (Figure S6A); Phred scores of majority reads are around 38 (Figure S6B); there were about 10% reads (<18 nts and >25 nts) that were discarded (Figure S6C); after processing, mapping rate to the rat genome ranged from 60 to 80% (Figure S6D); average mapping rate against to miRBase (released June. 2014) is 50% (Figure S6E); in total, 664 miRNAs have at least one read mapped with at least one sample. Low abundant miRNA sequences containing less than five normalized reads per million were removed. The removal of low abundant miRNA reads eliminates possible artifacts obtained from the normalization of miRNA that is not present in one or more samples. Therefore, miRNAs with high read counts are considered abundant and consists of actual raw reads before and after post-processing of NGS data. To evaluate the consistency of data sets among three replicates (rats), we performed scatterplot analyses to estimate the correlation coefficients at low, middle and high doses during 3-, 7-, 14- and 28-d. Figures S3–5 provide detailed scatterplots of the low-, middle- and high-dose exposures.

### Differentially expressed miRNA (DEM) analysis

The miRDeep2 pipeline was applied for the mapping of the reads to miRBase Release 21^48, 49^ and the quantification. Reads per million and upper quartile normalization were performed to adjust the library size and RNA composition. Details about this normalization method can be found in Rapaport, *et al*.^50^. DEMs was determined using edgeR^51^ with a threshold of fold change >1.5 and p-value < 0.05.

### Time and dose-response analysis

To identify time- and/or dose-dependent expression changes in miRNAs, all DEMs were processed and compiled into a single file: a holistic view of deregulated miRNAs. This file was then utilized to generate Venn diagrams to find out the overlapping as well as condition specific (dose or time) miRNAs among all the treated samples. An additional analysis was also accomplished to determine the time and dose response trends in overlapping DEMs. Changes in miRNA level were considered as time responsive if its relative expression level (fold change) follows an increasing or a decreasing pattern along the four time-points (3-d, 7-d, 14-d and 28-d) in at least two dose levels. Similarly, changes in miRNA levels were considered as dose-responsive if its relative expression level increases or decreases along the three doses (low, middle and high) in at least two-time points.

### Target genes identification and functional analysis of DEMs

Two different approaches were adopted to determine target genes of DEMs and their functional interpretation. In the first approach, the Ingenuity Pathway Analysis (IPA) (http://www.ingenuity.com) software package was used to analyze DEMs and their target genes using the miRNA/mRNA target analysis functionality. Experientially observed target genes for DEMs were retrieved from IPA. Disease enrichment analysis of miRNAs and functional pathway enrichment of experientially observed target genes were performed with IPA. Molecular networks were generated to graphically display the interconnected miRNAs and their target genes. In the second approach, information on putative target genes of DEMs was downloaded from miRWalk2.0^52, 53^ using seven different prediction algorithms. These miRNA-gene interactions were further filtered by applying the intersection criterion in which only those target genes that qualify for pathway enrichment analyses which are predicted with at least 2 out of 7 algorithms^54–56^. This filtered information was interrogated against the gene sets of KEGG (Kyoto Encyclopedia of Genes and Genomes) pathways. A customized script was executed in R-statistical package (R x3.2.4) environment which estimates the statistical enrichment of signaling cascades associated with the target genes of DEMs with a cut-off of 5% from Fisher’s exact test and multiple test adjustment using the Benjamini and Holm (BH) method^54, 55, 57, 58^.

### qRT-PCR validation

Custom-made TaqMan® Low-Density Array (TLDA) cards for miRNA expression analysis (Applied Biosystems, Life Technologies, Carlsbad, CA, USA) were used for quantification of specific miRNAs, each card allowing 384 simultaneous qPCR reactions of two different miRNAs in duplicates. U6 was used as reference gene and one mandatory control. Ten nanograms of total RNA was used to generate cDNA with the TaqMan microRNA reverse transcription kit (Applied Biosystems). TaqMan miRNA primer probes (Applied Biosystems) specific for mature miRNA were used for RT-PCR on a Viia7 PCR and detection instrument. Samples were normalized using the endogenous U6 control gene. Unpaired two-tailed Student’s t-tests were performed to determine statistically significant differences in miRNA expression in treated rats compared to the control.

### Histopathological data acquisition

The histopathological features of the rat livers treated with TAA were gathered from TG-GATE database to perform a regression analysis. These histopathological features are carefully determined by expert pathologists on the liver tissues treated with 3 doses (low, middle and high) and 4 time points (3-d, 7-d, 14-d, and 28-d). These features are nuclear alteration, cellular foci, cellular infiltration, change in eosinophilic, degeneration of granular eosinophilic, fibrosis, hypertrophy, bile duct proliferation, proliferation of ova cell, single cell necrosis (Figure S1). In order to carry out a regression analysis between expression data and histopathological features, the data points for 3-d, 7-d and 14-d with low dose exposures were removed because no histopathological changes were observed at these time points. 1.5-fold change was applied as a cutoff to generate a confidence matrix. This matrix was then imported into MATLAB (version R2016a) and “perfcurve” function was applied to calculate area under the curve (AUC) with the help of receiver operating characteristic (ROC).

### MicroRNAs Sequencing Data Availability Statement

The datasets generated and analyzed during the current study are available in the GEO repository (GSE87446). The following link has been created to allow review of record GSE87446 while it remains in private status: https://www.ncbi.nlm.nih.gov/geo/query/acc.cgi?token=gpevyqegphgxbwb&acc=GSE87446. Upon acceptance of this manuscript, the dataset will be opening to the public.

## Electronic supplementary material

Supplemental information

Supplementary Table S1

Supplementary Table S2

Supplementary Table S3

Supplementary Table S4

Supplementary Table S5

## References

[1] Obiri, S. *et al*. Human Health Risk Assessment of Artisanal Miners Exposed to Toxic Chemicals in Water and Sediments in the PresteaHuni Valley District of Ghana. *Int J Environ Res Public Health* **13** (2016). 10.3390/ijerph13010139 PMC4730530 26797625

[2] Tsuji JS, Garry MR, Perez V, Chang ET (2015). Low-level arsenic exposure and developmental neurotoxicity in children: A systematic review and risk assessment. Toxicology.

[3] Zerdoumi Y (2015). A new genotoxicity assay based on p53 target gene induction. Mutat Res Genet Toxicol Environ Mutagen.

[4] Ravegnini G, Sammarini G, Hrelia P, Angelini S (2015). Key Genetic and Epigenetic Mechanisms in Chemical Carcinogenesis. Toxicol Sci.

[5] Labib, S. *et al*. Comparative transcriptomic analyses to scrutinize the assumption that genotoxic PAHs exert effects via a common mode of action. *Arch Toxicol* (2015). 10.1007/s00204-015-1595-5 PMC5043007 26377693

[6] Kirkland D, Reeve L, Gatehouse D, Vanparys P (2011). A core *in vitro* genotoxicity battery comprising the Ames test plus the *in vitro* micronucleus test is sufficient to detect rodent carcinogens and *in vivo* genotoxins. Mutat Res.

[7] Parry JM, Parry E, Phrakonkham P, Corvi R (2010). Analysis of published data for top concentration considerations in mammalian cell genotoxicity testing. Mutagenesis.

[8] Benigni R, Bossa C, Tcheremenskaia O (2013). Nongenotoxic carcinogenicity of chemicals: mechanisms of action and early recognition through a new set of structural alerts. Chem Rev.

[9] Boffetta P, Islami F (2013). The contribution of molecular epidemiology to the identification of human carcinogens: current status and future perspectives. Ann Oncol.

[10] Eichner J, Wrzodek C, Romer M, Ellinger-Ziegelbauer H, Zell A (2014). Evaluation of toxicogenomics approaches for assessing the risk of nongenotoxic carcinogenicity in rat liver. PLoS One.

[11] Beland FA, Olson GR, Mendoza MC, Marques MM, Doerge DR (2015). Carcinogenicity of glycidamide in B6C3F1 mice and F344/N rats from a two-year drinking water exposure. Food Chem Toxicol.

[12] Ding W (2012). *In vivo* genotoxicity of furan in F344 rats at cancer bioassay doses. Toxicol Appl Pharmacol.

[13] Sistare FD (2011). An analysis of pharmaceutical experience with decades of rat carcinogenicity testing: support for a proposal to modify current regulatory guidelines. Toxicol Pathol.

[14] Huff J, Jacobson MF, Davis DL (2008). The limits of two-year bioassay exposure regimens for identifying chemical carcinogens. Environ Health Perspect.

[15] Seely JE, Pegg AE (1983). Effect of 1,3-diaminopropane on ornithine decarboxylase enzyme protein in thioacetamide-treated rat liver. Biochem J.

[16] Sheikh, T.A.a.N. An overview of thioacetamide-induced hepatotoxicity. *Informa* **32** (2013).

[17] Chapman KL (2013). Pharmaceutical toxicology: designing studies to reduce animal use, while maximizing human translation. Regul Toxicol Pharmacol.

[18] Black MB (2015). Using gene expression profiling to evaluate cellular responses in mouse lungs exposed to V2O5 and a group of other mouse lung tumorigens and non-tumorigens. Regul Toxicol Pharmacol.

[19] Doktorova TY (2012). Comparison of hepatocarcinogen-induced gene expression profiles in conventional primary rat hepatocytes with *in vivo* rat liver. Arch Toxicol.

[20] Hoenerhoff MJ (2011). Global gene profiling of spontaneous hepatocellular carcinoma in B6C3F1 mice: similarities in the molecular landscape with human liver cancer. Toxicol Pathol.

[21] Thomas RS (2012). Integrating pathway-based transcriptomic data into quantitative chemical risk assessment: a five chemical case study. Mutat Res.

[22] Zhong J, Chen Y, Wang LJ (2016). Emerging molecular basis of hematogenous metastasis in gastric cancer. World J Gastroenterol.

[23] Prasadam, I. *et al*. Systematic Identification, Characterization and Target Gene Analysis of microRNAs Involved in Osteoarthritis Subchondral Bone Pathogenesis. *Calcif Tissue Int* (2016). 10.1007/s00223-016-0125-7 26944279

[24] Lewis, S. MicroRNA gets motoring. *Nature Reviews Neuroscience* **15** (2014). 10.1038/nrn3672 24370875

[25] Paul Graves, Y. Z. Biogenesis of Mammalian MicroRNAs: A Global View. *Genomics, Proteomics & Bioinformatics* **10** (2012). 10.1016/j.gpb.2012.06.004 PMC5054211 23200133

[26] Abue M (2015). Circulating miR-483-3p and miR-21 is highly expressed in plasma of pancreatic cancer. Int J Oncol.

[27] Eminaga S1, C. D., Vigneault, F., Church, G. M., Seidman, J. G. Quantification of microRNA expression with next-generation sequencing. *Curr Protoc Mol Biol*. Jul (2013). 10.1002/0471142727.mb0417s103 PMC4138881 23821442

[28] Nekrutenko A, Taylor J (2012). Next-generation sequencing data interpretation: enhancing reproducibility and accessibility. Nature Reviews Genetics.

[29] Hailey JR (2014). Biliary proliferative lesions in the Sprague-Dawley rat: adverse/non-adverse. Toxicol Pathol.

[30] Yeh CN, Maitra A, Lee KF, Jan YY, Chen MF (2004). Thioacetamide-induced intestinal-type cholangiocarcinoma in rat: an animal model recapitulating the multi-stage progression of human cholangiocarcinoma. Carcinogenesis.

[31] Liu Z, Wang Y, Borlak J, Tong W (2016). Mechanistically linked serum miRNAs distinguish between drug induced and fatty liver disease of different grades. Sci Rep.

[32] Keller A, Meese E (2016). Can circulating miRNAs live up to the promise of being minimal invasive biomarkers in clinical settings?. Wiley Interdiscip Rev RNA.

[33] Liu W, Cao H, Yan J, Huang R, Ying H (2015). ‘Micro-managers’ of hepatic lipid metabolism and NAFLD. Wiley Interdiscip Rev RNA.

[34] Li Z (2010). Genomic analysis of microRNA time-course expression in liver of mice treated with genotoxic carcinogen N-ethyl-N-nitrosourea. BMC Genomics.

[35] Liu LN, Li DD, Xu HX, Zheng SG, Zhang XP (2015). Role of microRNAs in hepatocellular carcinoma. Front Biosci (Landmark Ed).

[36] Wang L, Yue Y, Wang X, Jin H (2015). Function and clinical potential of microRNAs in hepatocellular carcinoma. Oncol Lett.

[37] Morishita A, Masaki T (2015). miRNA in hepatocellular carcinoma. Hepatol Res.

[38] Basu, S. & Murphy, M. E. Genetic Modifiers of the p53 Pathway. *Cold Spring Harb Perspect Med* **6** (2016). 10.1101/cshperspect.a026302 PMC4817744 27037420

[39] Harris SL, Levine AJ (2005). The p53 pathway: positive and negative feedback loops. Oncogene.

[40] Li X (2015). microRNA-34a and microRNA-34c promote the activation of human hepatic stellate cells by targeting peroxisome proliferator-activated receptor gamma. Mol Med Rep.

[41] Xiao Z (2014). A small-molecule modulator of the tumor-suppressor miR34a inhibits the growth of hepatocellular carcinoma. Cancer Res.

[42] Chalanqui, M. J., O’Doherty, M., Dunne, N. J. & McCarthy, H. O. MiRNA 34a: a therapeutic target for castration-resistant prostate cancer. *Expert Opin Ther Targets* 1–11 (2016). 10.1517/14728222.2016.1162294 26942553

[43] Guicciardi ME, Malhi H, Mott JL, Gores GJ (2013). Apoptosis and necrosis in the liver. Compr Physiol.

[44] Shibata C (2013). Inhibition of microRNA122 decreases SREBP1 expression by modulating suppressor of cytokine signaling 3 expression. Biochem Biophys Res Commun.

[45] Li A (2013). Modulation of miR122 expression affects the interferon response in human hepatoma cells. Mol Med Rep.

[46] Hsu SH (2012). Essential metabolic, anti-inflammatory, and anti-tumorigenic functions of miR-122 in liver. J Clin Invest.

[47] Uehara, T. *et al*. Prediction model of potential hepatocarcinogenicity of rat hepatocarcinogens using a large-scale toxicogenomics database. *Toxicology and Applied Pharmacology* **255** (2011). 10.1016/j.taap.2011.07.001 21784091

[48] Kozomara A, Griffiths-Jones S (2011). miRBase: integrating microRNA annotation and deep-sequencing data. Nucleic Acids Research.

[49] Kozomara, A. & G.-J.S. Annotating high confidence microRNAs using deep sequencing data. *Nucleic Acids Res* **42** (2014). 10.1093/nar/gkt1181 PMC3965103 24275495

[50] Rapaport, F., K. R. & Liang, Y. Comprehensive evaluation of differential gene expression analysis methods for RNA-seq data. *Genome Biology* **14** (2013). 10.1186/gb-2013-14-9-r95 PMC4054597 24020486

[51] Robinson, M. D., M. D. & Smyth, G. K. edgeR: a Bioconductor package for differential expression analysis of digital gene expression data. *Bioinformatics* **26** (2010). 10.1093/bioinformatics/btp616 PMC2796818 19910308

[52] Dweep H, Gretz N (2015). miRWalk2.0: a comprehensive atlas of microRNA-target interactions. Nat Methods.

[53] Dweep H, Sticht C, Pandey P, Gretz N (2011). miRWalk–database: prediction of possible miRNA binding sites by “walking” the genes of three genomes. J Biomed Inform.

[54] Dweep H (2013). CNVs-microRNAs interactions demonstrate unique characteristics in the human genome. An interspecies *in silico* analysis. PLoS One.

[55] Gaynullina D (2015). Alteration of mRNA and microRNA expression profiles in rat muscular type vasculature in early postnatal development. Sci Rep.

[56] Qi X (2014). Ochratoxin A induced early hepatotoxicity: new mechanistic insights from microRNA, mRNA and proteomic profiling studies. Scientific Reports.

[57] Dweep H, Kubikova N, Gretz N, Voskarides K, Felekkis K (2015). Homo sapiens exhibit a distinct pattern of CNV genes regulation: an important role of miRNAs and SNPs in expression plasticity. Sci Rep.

[58] Dweep H, Sticht C, Kharkar A, Pandey P, Gretz N (2013). Parallel analysis of mRNA and microRNA microarray profiles to explore functional regulatory patterns in polycystic kidney disease: using PKD/Mhm rat model. PLoS One.

