# Supplementary material for: Mechanistic roles of microRNAs in hepatocarcinogenesis: A study of thioacetamide with multiple doses and time-points of rats
Source: Sci Rep. 2017 Jun 8;7:3054. doi: 10.1038/s41598-017-02798-7 (PMC5465221; doi:10.1038/s41598-017-02798-7)
Supplement: Supplementary file 1 — Supplemental information [file 41598_2017_2798_MOESM1_ESM.pdf]

Supplement to

Mechanistic roles of microRNAs in hepatocarcinogenesis: A study of thioacetamide with multiple doses and time-points of rats

Harsh Dweep[1]*, Yuji Morikawa[2]*, Binsheng Gong[1]*, Jian Yan[3], Zhichao Liu[1], Tao Chen[3], Halil Bisgin[5], Wen Zou[1], Huixiao Hong[1], Tieliu Shi[6], Ping Gong[7], Christina Castro[4], Takeki Uehara[8#], Yuping Wang[1#], Weida Tong[1#]

Supplementary Figure S1. Overview of the histopathological features of the rat liver (treated with TAA) observed by expert pathologists and documented under TG-GATE database. The three columns describe the histopathological features, AUC values for miR-34a-5p and miR-455-3p. The columns (4-15) header contain information on dose and time points, for example, “Low_3-d” stands for low dose at 3 day. The “green” and “grey” colors denote a feature is “found” and not “found”, respectively.

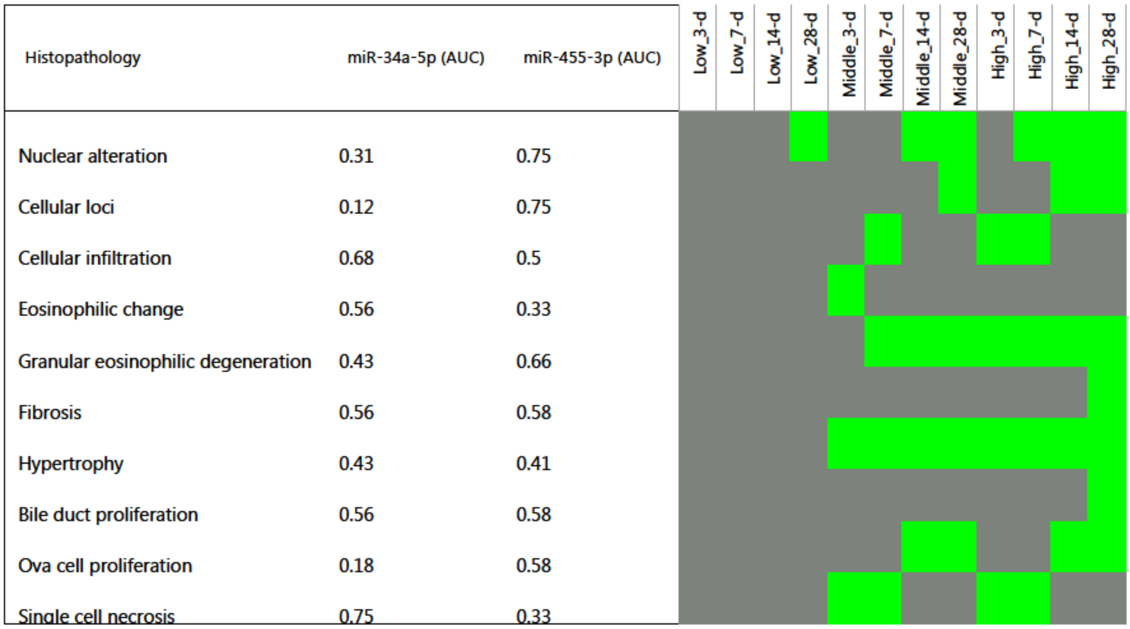

Supplementary Figure S2. qRT-PCR validation of rno-miR-34a-5p at high dose with four time-points. The figure shows a high abundance of transcripts of rno-miR-34a-5p in TAA treated (high dose) with respect to control liver samples. '*' indicates a significant difference of CT and fold change values in treated rats compared to the control group. X-axis describes time intervals of high-dose treatment and y-axis depicts CT values.

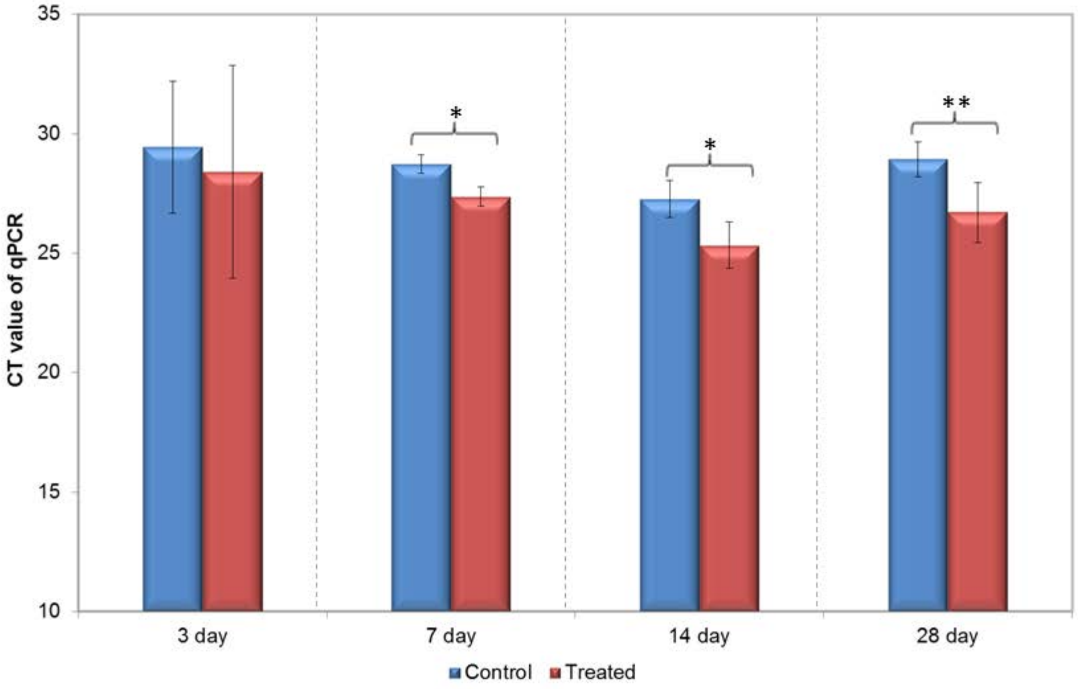

Supplementary Figure S3. Scatterplots of all pairwise comparisons of log2 values among all three replicates during the entire course of low dose exposure treatments (3-, 7-, 14- and 28-d). The upper and lower triangles of each square show the log2 values along with regression estimation bisector lines and correlation coefficient among each pair. The “***” indicates high significance probability values ($p<0.001$). Each sample name stands for a rat treated with thioacetamide low dose, for example, “L3_rat1” depicts low dose at 3 day in rat 1. The average correlation values among the three rats are approximately 0.98 which indicate a great consistent among the data sets used for elucidating the role of miRNAs in hepatocarcinogenicity.

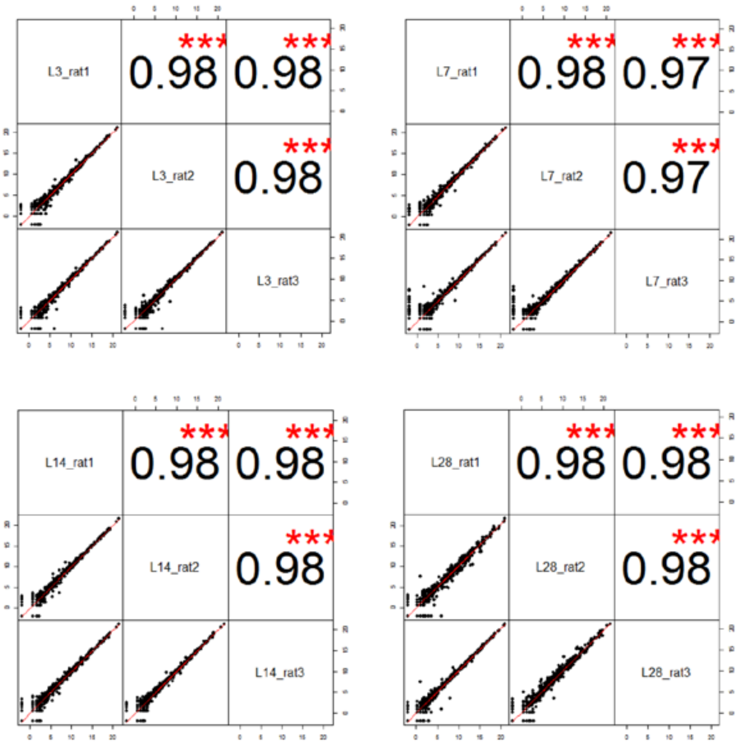

Supplementary Figure S4. Scatterplots of all pairwise comparisons of log2 values among all three replicates during the entire course of middle dose exposure treatments (3-, 7-, 14- and 28-d). The upper and lower triangles of each square show the log2 values along with regression estimation bisector lines and correlation coefficient among each pair. The “***” indicates high significance probability values ($p<0.001$). Each sample name stands for a rat treated with thioacetamide middle dose, for example, “M14_rat3” depicts middle dose at 14 day in rat 3. The average correlation values among the three rats are approximately 0.98 which indicate a great consistent among the data sets used for elucidating the role of miRNAs in hepatocarcinogenicity.

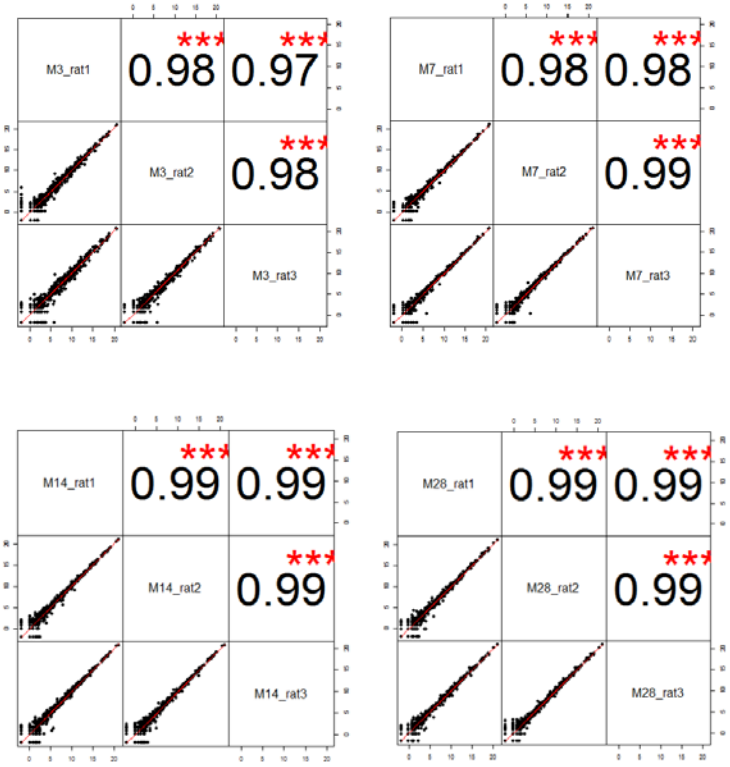

Supplementary Figure S5. Scatterplots of all pairwise comparisons of log2 values among all three replicates during the entire course of high dose exposure treatments (3-, 7-, 14- and 28-d). The upper and lower triangles of each square show the log2 values along with regression estimation bisector lines and correlation coefficient among each pair. The "***" indicates high significance probability values (p<0.001). Each sample name stands for a rat treated with thioacetamide high dose, for example, "H28_rat2" depicts high dose at 28 day in rat 2. The average correlation values among the three rats are approximately 0.98 which indicate a great consistent among the data sets used for elucidating the role of miRNAs in hepatocarcinogenicity.

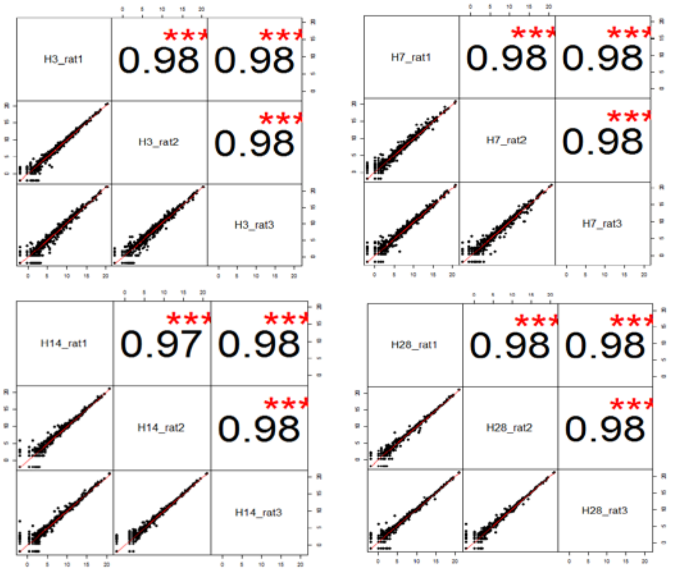

Supplementary Figure S6. Overview of quality control and assessment of NGS data.

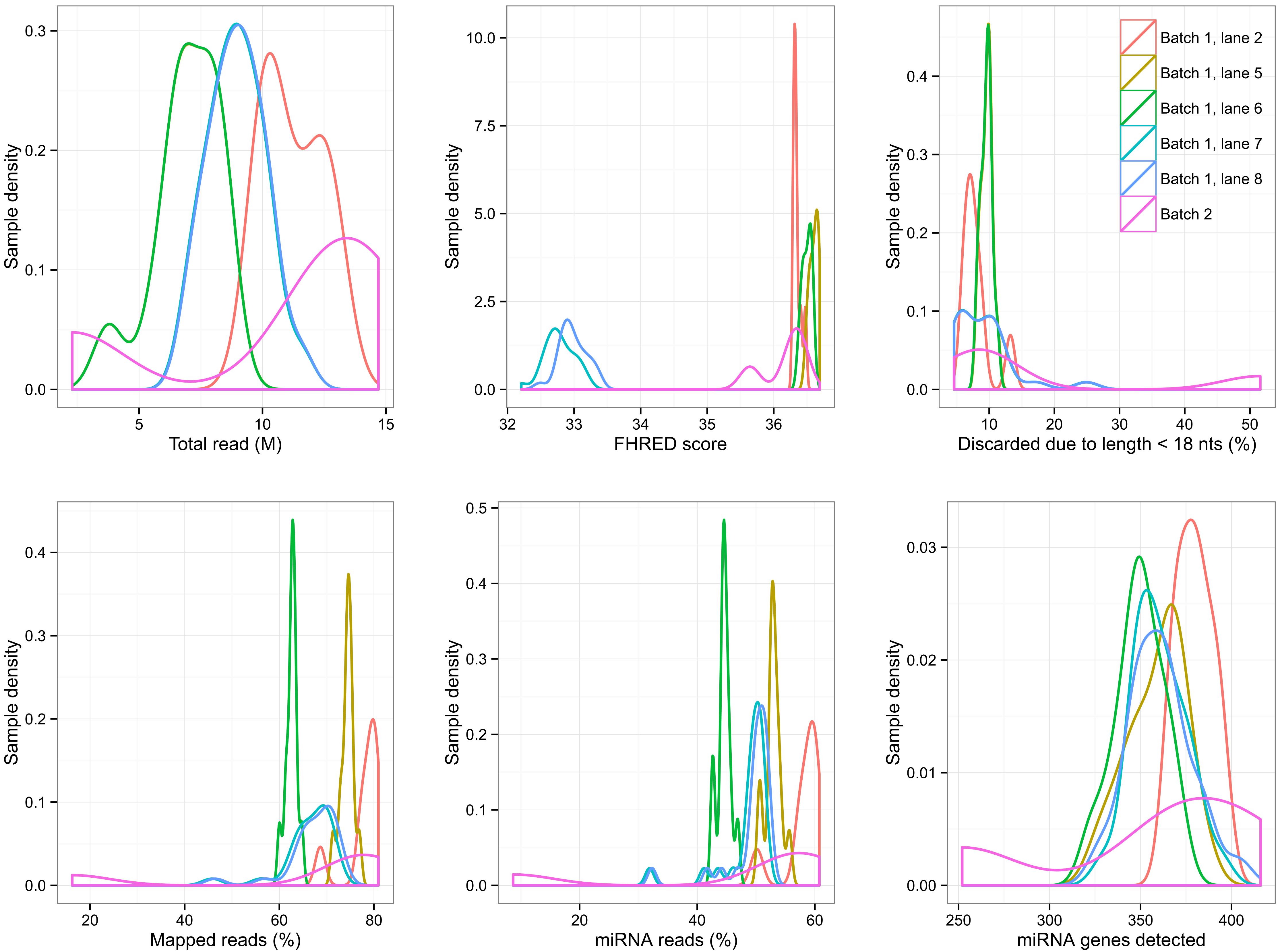
Sample density
Total read (M)
FHRED score
Discarded due to length < 18 nts (%)
Batch 1, lane 2
Batch 1, lane 5
Batch 1, lane 6
Batch 1, lane 7
Batch 1, lane 8
Batch 2
Mapped reads (%)
miRNA reads (%)
miRNA genes detected

Supplementary Tables 1-5

Table S1. Differentially regulated miRNAs identified during TAA treatment with different doses and time points.

Table S2. Significantly enriched diseases obtained on differently expressed miRNAs during different treatment conditions.

Table S3. Significantly enriched pathways obtained on experimentally verified target genes of deregulated miRNAs during different doses and time points using IPA software.

Table S4. Significantly enriched pathways found on putative target genes of up-regulated miRNAs using miRWalk2.0.

Table S5. Overrepresented pathways determined on putative target genes of down-regulated miRNAs using miRWalk2.0.
